# Labyrinthine Bifurcation of the Facial Nerve

**DOI:** 10.1007/s12070-023-03977-x

**Published:** 2023-07-15

**Authors:** Miguel Saro-Buendía, Raúl Mellidez Acosta, Catalina Bancalari Díaz, Miguel Mazón, Carlos de Paula Vernetta, Miguel Armengot Carceller

**Affiliations:** 1 Servicio de Otorrinolaringología, Hospital Universitario y Politécnico La Fe, València, España; 2 Departament de Cirugia, Facultat de Medicina i Odontología, Universitat de València, València, España; 3 Servicio de Radiología, Hospital Universitario y Politécnico La Fe, València, España

**Keywords:** Bifurcation of the facial nerve, Labyrinthine bifurcation, Congenital malformations of the facial nerve, Congenital temporal bone anomalies, Facial nerve anatomical variations, Congenital aural atresia

## Abstract

The labyrinthine bifurcation of the facial nerve is extremely rare. Diverse congenital temporal bone anomalies usually coexist, and a detailed preoperative evaluation is needed to detect them. We report a case of labyrinthine bifurcation of the facial nerve detected on the preoperative evaluation of a patient with congenital aural atresia.

## Introduction

Congenital malformations of the facial nerve (FN) are uncommon [1]. Bifurcation of the FN is specially rare, but has been described in all its segments [1]. The most common bifurcation affects the tympanic segment, typically above the oval window [1]. However, only one case has been described in the labyrinthine segment [1, 2]. Then, our aim is to report the second case of labyrinthine bifurcation of the FN to aware physicians on this malformation and its clinical implications.

## Case Report

A 3-year-old female, with family history of Nager dysostoses (maternal cousin), presented to our centre with hypoacusis and facial dysostoses. Additionally, she presented a bilateral congenital aural atresia (CAA), micrognathia, microtia (grade I) and mandibular synodontia. However, she had passed the hearing screening at birth.

We performed a conditioned play audiometry; it showed a bilateral conductive hearing loss with air- conduction thresholds at 60–70 dB. Genetic test for dysmorphias was negative. However, a new alteration was found (2q11.1q11.2).

**Fig. 1:**
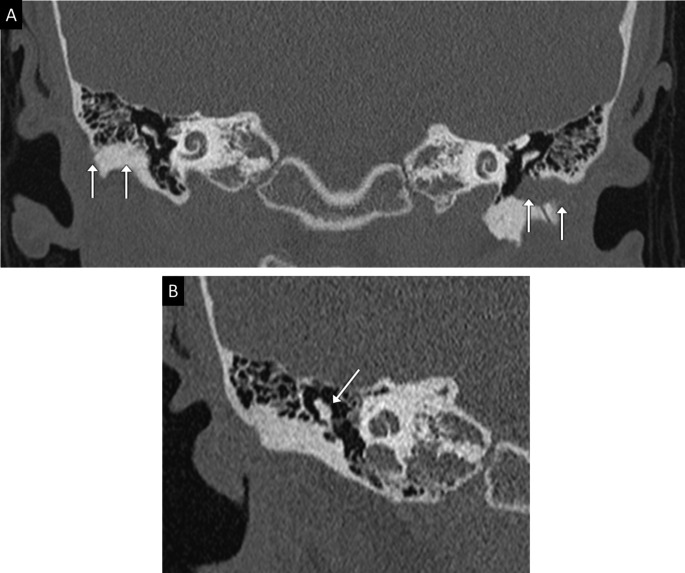
Computed tomography (coronal views) focused on the external and middle ear structures. **(a)** Bilateral complete aural atresia. It is fibro-osseous on the right side and fibrous on the left side (arrows). **(b)** Right malleolar dysplasia, the malleus is fused with the lateral wall of the tympanic cavity (arrows)

**Fig. 2:**
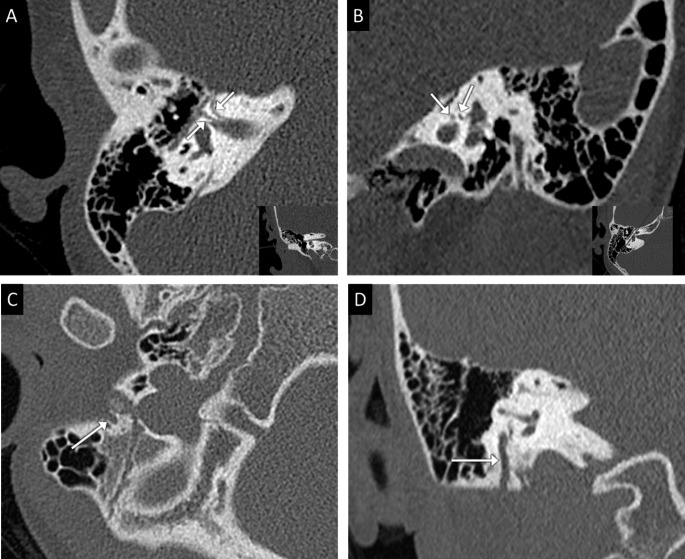
Computed tomography focused on the right intratemporal facial nerve. **(a)** axial oblique view, **(b)** view perpendicular to the labyrinthine segment: Right facial nerve bifurcation on its labyrinthine segment (arrows). **(c)** axial view, **(d)** coronal view: short and anterior mastoid segment of the facial nerve opened anteriorly between the mastoid apophysis and the glenoid cavity (arrows)

A temporal bone computed tomography (CT) identified a bilateral complete aural atresia (Fig. 1a) and a right malleolar dysplasia (Fig. 1b). Also were described a bifurcation of the labyrinthine segment of the right FN (Fig. 2a and b) and an ipsilateral short and anterior mastoid segment (Fig. 2c and d).

Since the third year of life (since she visited our centre), non-surgical bone conduction hearing aids were adapted bilaterally. One year later, a passive transcutaneous bone anchored hearing device was implanted on the right ear. Currently, the patient is 9 years old and a left side canalplasty with ossiculoplasty is to be performed soon.

## Discussion

The diverse FN anatomical variations and their relationship with middle and external ear anomalies are explained by a complex embryologic process, extended from the third week of gestation until the postnatal period [3]. The facioacoustic neural crest cells, present from the third week of gestation, are the origin of the FN. By the fifth week, the distal segment of the facial primordium is divided: one branch is directed towards the second branchial arch mesenchyme (future main trunk of the FN) and the other branch crosses the first branchial arch (future chorda tympani). By the eighth week, the membranous labyrinth is formed and the anatomic relation between the FN and other middle ear structures is well established. However, it is not until the fifth month of gestation that the temporal bone begins to cover the FN, this process finalizes postnatally [3]. The lack of bony covering (known as bony dehiscence of the FN) is very common in the tympanic segment and is considered a normal anatomical variation [4, 5].

The embryologic process explains the fact that bifurcation of the FN (as other congenital FN malformations) is usually coexistent with external, middle, or inner ear dysplasia [1, 2, 6]. In our case, there is a right FN bifurcation (labyrinthine segment) with an ipsilateral short and anterior mastoid segment. It is associated with bilateral congenital aural atresia (CAA) and a right malleolar dysplasia. CAA is due to a failure in the development of the external auditory canal (EAC). This structure arises from the fist branchial cleft since the second month of gestation and its canalization begins after the sixth month of gestation [6]. CAA is usually sporadic and often associates middle ear dysplasia and auricle abnormalities (like malleolar dysplasia and microtia in our case). Ossicles arise from the first and second branchial arches and their development takes place between the 4th and 16th weeks of gestation.

An early auditory brainstem response is useful to evaluate the auditory pathway and CT is useful to evaluate the thickness and composition of the atresia and to detect related anomalies. The intratemporal FN can be depicted on MRI and CT while distally to the mastoid segment indirect landmarks are needed to understand the FN course [7]. In our case a CT was enough to understand the CAA features, evaluate the middle ear structures and detect the FN anomalies.

In bilateral CAA, hearing amplification during the early years (including bone- anchored hearing devices) is essential to allow the development of speech and language [6]. Our experience is in accordance with this fact. We adapted non-surgical bone conduction hearing aids after her first visit (she was 3 years-old) and a year later we implanted a passive transcutaneous bone anchored hearing device on the right ear. The surgical approach of CAA is very challenging and in bilateral cases should be delayed until the age of 6–7 years-old [6]. The procedure generally includes canalplasty, tympanoplasty and meatoplasty [6]. Additional anatomical anomalies (like those present in our case) should be addressed to achieve optimal audiologic outcomes and minimize the likelihood of iatrogenic FN injury [8]. Even after basic tympanomastoidectomy procedures, iatrogenic FN injury is observed in 0.6–3.7% of the cases (4–10% after revision surgery) [9, 10]. In our case, canalplasty should be followed by an ossiculoplasty to achieve optimal audiologic outcomes. Also, a meticulous approach, including FN monitorization, will be needed to do not injure the FN at its anteriorly placed mastoid segment.

To summarize, we report the first case of labyrinthine bifurcation of the FN since it was described. This anomaly, like other congenital FN anomalies, may be associated to external and middle ear malformations due to common embryologic pathways. Once an otologic malformation has been detected, the temporal bone should be carefully evaluated to detect additional anomalies as they might have therapeutic implications. We report a case of labyrinthine bifurcation of the FN to familiarize physicians with this rare temporal bone condition.

## Abbreviations

FN: Facial nerve
CAA: Congenital aural atresia
CT: Computed tomography
EAC: External auditory canal

## References

[1] Gupta S, Mends F, Hagiwara M, Fatterpekar G, Roehm PC (2013). Imaging the facial nerve: a contemporary review. Radiol Res Pract.

[2] Glastonbury CM, Fischbein NJ, Harnsberger HR, Dillon WP, Kertesz TR (2003) *Congenital Bifurcation of the Intratemporal Facial Nerve Case Reports Case 1 From the Department of Radiology (C*. Vol 24.; PMC7973663 12917123

[3] Sataloff RT (1990). Embryology of the facial nerve and its clinical applications. Laryngoscope.

[4] Jahrsdoerfer RA (1981). The facial nerve in congenital middle ear malformations. Laryngoscope.

[5] Gershon Spector J (1993). Ossification patterns of the tympanic facial canal in the human fetus and neonate. Laryngoscope.

[6] Abdel-Aziz M (2013). Congenital aural atresia. J Craniofac Surg.

[7] Chhabda S, Leger DS, Lingam RK (2020) Imaging the facial nerve: a contemporary review of anatomy and pathology. Eur J Radiol 126. 10.1016/j.ejrad.2020.108920 10.1016/j.ejrad.2020.108920 32199143

[8] Lambert PR (1998) *Congenital Aural Atresia: Stability of Surgical Results*.; 10.1097/00005537-199812000-00007 9851494

[9] Hohman MH, Hadlock TA (2014) Etiology, diagnosis, and management of facial palsy: 2000 patients at a facial nerve center. Laryngoscope, vol 124. John Wiley and Sons Inc. doi:10.1002/lary.24542 10.1002/lary.24542 24431233

[10] Hohman MH, Bhama PK, Hadlock TA (2014). Epidemiology of iatrogenic facial nerve injury: a decade of experience. Laryngoscope.

